# Character Strengths Lead to Satisfactory Educational Outcomes Through Strength Use: A Longitudinal Analysis

**DOI:** 10.3389/fpsyg.2019.01829

**Published:** 2019-08-27

**Authors:** Xiaoqing Tang, Yumei Li, Wenjie Duan, Wenlong Mu, Xinfeng Cheng

**Affiliations:** ^1^School of Philosophy, Zhongnan University of Economics and Law, Wuhan, China; ^2^School of Sociology, Wuhan University, Wuhan, China; ^3^School of Social & Public Administration, East China University of Science and Technology, Shanghai, China; ^4^School of Economics and Management, Xi’an Technological University, Xi’an, China; ^5^School of Economics and Management, Wuhan University, Wuhan, China

**Keywords:** character strengths, strengths use, academic achievement, eudaimonic well-being, positive education, positive development, adolescents, longitudinal analysis

## Abstract

Despite the flourishing of positive education, understanding of whether different character strengths have different predictive effects on academic achievement/well-being and the mechanisms of actions between character strengths are limited. Specifically, this study adopted strength use as a mediator to understand how character strength (assessed by caring, inquisitiveness, and self-control) is associated with students’ end-of-year academic achievements and eudaimonic well-being. Survey data from 349 adolescents from three different schools showed that three factors of character strengths have positive correlations with academic achievements and eudaimonic well-being. Regression models indicated that inquisitiveness and self-control predicted academic achievements, while caring, inquisitiveness, and self-control predicted eudaimonic well-being, with the foremost as the strongest predictor. Mediation analyses indicated that (1) strengths use fully mediated the relationship between inquisitiveness, self-control, and academic achievements/eudaimonic well-being, while (2) caring had a direct effect on eudaimonic well-being. These findings provided possible explanations on how character strengths could affect students’ academic achievements or eudaimonic well-being and theoretical and empirical evidence for practices that aim to enhance students’ academic achievements and positive developments via interventions based on character strengths.

## Introduction

In recent years, the flourishing of positive psychology has corrected the imbalances in traditional psychological research and practices. Positive psychology deals with the positive aspects of human functioning (e.g., courage, perseverance, forgiveness, and originality), valued experience (e.g., well-being, contentment, satisfaction, and hope), civic virtues, and the institutions that move individuals toward good citizenship (e.g., responsibility, altruism, civility, and work ethic) (Seligman and Csikszentmihalyi, 2000). The core theme of positive psychology is “character strengths” (Peterson and Seligman, 2004), which can be conceptualized as the positive qualities or dispositions of interests, talents, values, emotions, and thoughts manifested by individuals (Park and Peterson, 2009).

An increasing number of psychologists and educators suggest the application of positive psychology elements in this field to eliminate the negative tendency in traditional education, enhance the balance in education, and deepen its essence (Seligman et al., 2009; Shoshani and Steinmetz, 2014; Shoshani and Slone, 2017). Unlike traditional education that focuses on accomplishments, positive education proposes that accomplishments (e.g., academic achievements) and well-being are equally important components in the development of students’ life outcomes (International Positive Education Network, 2019). Well-being emphasizes individuals’ positive functioning, including competence, engagement, meaning and purpose, optimism, self-acceptance, supportive relationships, well-being of others, and being respected (Ryff and Keyes, 1995; Diener et al., 2010; Duan and Xie, 2019). Positive education advocates that character strengths is a potential pathway to facilitate students’ academic achievements and well-being in the educational context (Seligman et al., 2009; International Positive Education Network, 2019).

Peterson and Seligman (2004) developed the Values in Action (VIA) classification as a systematic framework to study character strengths. However, their two-tier structure of six virtues and 24 character strengths was unsupported by most empirical studies (e.g., Macdonald et al., 2008; Duan et al., 2012; Azañedo et al., 2014) with one exception (Ruch and Proyer, 2015). These results suggest that an alternative factor structure of character strengths can be developed. After the careful controlling of cultural influence, a few scholars have independently found the effectiveness of 24 character strengths grouped into a three-factor structure using various samples and instruments (e.g., Duan et al., 2012; McGrath, 2015; Duan and Bu, 2017; Duan and Ho, 2017; Park et al., 2017), namely, caring, inquisitiveness, and self-control. Caring indicates the strengths (e.g., kindness, authenticity, and fairness) involved in maintaining agreeable relations toward others, inquisitiveness indicates the strengths (e.g., curiosity, creativity, and zest) that describe the curiosity and creativity to associate oneself with all life in the world, and self-control indicates the strengths (e.g., self-regulation, judgment, and love of learning) reflecting the regulation and adaptation ability in achieving values and goals (Duan and Bu, 2017). McGrath (2019) implied that the three-factor model is a reliable latent structure of the VIA classification form from cultural and psychological perspectives.

Extant studies have demonstrated that character strengths were positively related to the cognitive and emotional types of educational outcomes, including academic achievements (Lounsbury et al., 2009; Kern and Bowling, 2015) and well-being (Tang et al., 2016; Hausler et al., 2017). However, three dimensions of character strengths were found to have a different focus on academic achievements or well-being. For instance, although a study found that the intrapersonal, intellectual, and interpersonal strengths are positively correlated with grade point average above demographic covariates, only the intrapersonal strength was significant when predicting the growth of grade point average (Park et al., 2017). A longitudinal study that followed 417 students and 13 teachers from four public middle schools found that temperance and intellectual strength, rather than interpersonal strength, were central in the prediction of students’ academic performances and achievements (Shoshani and Slone, 2013). Notwithstanding, in a two-year study of high school students, only other-directed strengths (similar to caring strengths) were strong predictors of well-being when controlling the influence of the temperance and intellectual strengths (Gillham et al., 2011). In the framework of positive education, academic achievements, and well-being must be developed simultaneously, however, the abovementioned studies only separately discussed the relationship between character strengths and academic achievements or well-being. Identifying the profiles of strengths that are strongly linked to academic achievements and well-being is crucial.

More importantly, in line with Linley et al. (2006), exploring the processes and mechanisms that lead to valued educational outcomes should be one of the key issues of positive education. Peterson and Seligman (2004) emphasized that individuals with specific character strengths act in accordance with such strengths and be intrinsically motivated to use them. When people use their character strengths, they follow their own will and natural capacities to fulfill their potential and achieve their goals, which would lead to valued outcomes such as achievements and well-being (Linley and Harrington, 2006). Consequently, the Aware-Explore-Apply (A-E-A) model was proposed to describe how strength-based approaches lead to valued outcomes (Niemiec, 2013). The awareness phase aims to help individuals build the knowledge of their strengths, the exploring phase allows participants to understand how character strengths relate to valued outcomes through past and current experiences, and finally, the applying phase focuses on using character strengths in daily settings.

According to the A-E-A model, strength knowledge and strength use were two successive phases, with the former as the “launching point” (Shankland and Rosset, 2017). However, Zhang and Chen (2018) argued that if a person knows his character strengths well but never uses them, then he is unlikely to gain many benefits from these strengths. Therefore, strengths use is the direct route although strength knowledge is a prerequisite for obtaining valuable outcomes. Consistent with this, Quinlan et al. (2015) attempted a six-session intervention with 9- to 12-year-old students and found that rather than strength knowledge, strength use was associated with well-being changes and achievements. Thereafter, in a 1-year randomized controlled intervention Duan et al. (2018a) found that changes in strength use were significantly correlated with well-being, whereas changes in the awareness and recognition of character strengths did not significantly predict well-being. Thus, believing that strength use rather than strength knowledge was the working component is reasonable.

In sum, the above literature reviews imply the following. (a) Although general positive associations between character strengths and academic achievements/well-being are confirmed, character strengths may have different focuses when predicting educational outcomes. (b) Apart from exploring the direct relationship between variables, clarifying the processes, and mechanisms that lead to valued educational outcomes should be more important in positive education. From the perspective of developmental psychology, the cognitive style and behavioral habits formed in adolescence play a decisive role in future life developments because people experience more rapid growth changes during this period than in any other life phases, except infancy (Carnegie Council on Adolescent Development, 1989). Under educational psychology, education received in schools is effective in shaping adolescents’ cognitive styles and behavioral habits because they spend considerable time in these institutions (McLaughlin and Clarke, 2010). Therefore, the present study aims to address the abovementioned gaps by using longitudinal data from adolescents. Specifically, the following hypotheses are examined: (a) self-control and inquisitiveness strengths are stronger than caring when predicting academic achievements; (b) caring strengths are stronger than self-control and inquisitiveness when predicting well-being; and (c) strength use plays a mediating role between character strengths and valued educational outcomes, including academic achievements, and well-being.

To the best of our knowledge, this is the first study to examine the mediation role of strength use between the three-dimensional model of character strengths and educational outcomes among adolescents. The longitudinal design is used with the perspective of positive education. The results further clarify the key factors at work in the A-E-A model and then provide guidance for future effective strength interventions based on such model. Moreover, the findings can help educators develop effective strength-based positive education programs to improve students’ academic achievements and eudaimonic well-being, as well as provide enlightenment significance to conduct positive education projects for teachers and practitioners in the future.

## Materials and Methods

### Participants and Procedures

Participants included seventh to ninth grade students from three urban middle schools in Guangxi, Jiangsu, and Chongqing, China. All students in the said grade levels were welcome to participate in the present study. The three participating schools are all ordinary middle schools and were selected according to convenience. In each school, recruitment announcements were posted on the notice board 3 days before data collection. Data were collected through a paper-and-pencil method by trained master students majoring in social work. Parents and students signed consent forms before the latter completed the surveys.

The questionnaires were completed during the academic year. Different measures were used at each time point to test the predictive ability of character strengths. Character strengths and strength use were assessed at the beginning of the spring semester (February 2016) for the academic year 2015–2016 (Time 1). The order of scales was randomized to reduce any systematic order effect. Eudaimonic well-being was measured at the end of the same semester (July 2016), and the final exam grades were collected from the official school records (Time 2). In this survey, 359 students participated at Time 1, and 356 students participated at Time 2. Three students did not complete Time 2 because they transferred to another school. Seven students who forgot or missed to fill out all the items were removed. Finally, a total of 349 responses (193 females and 156 males, mean age = 13.64, *SD* = 0.94, age range = 12–17) were considered valid.

### Instruments

#### Three-Dimensional Inventory of Character Strengths (TICS)

Character strengths were measured using the self-reported 15-item TICS (Duan and Bu, 2017) on a five-point Likert scale (1 = “very much unlike me” to 5 = “very much like me”). Caring (e.g., “I enjoy being kind to others”), inquisitiveness (e.g., “I am always coming up with new ways to do things”), and self-control (e.g., “I am a highly disciplined person”) were identified in previous empirical studies (Duan et al., 2012; Duan and Ho, 2017). High mean scores indicate considerable character strengths. Prior research has showed an acceptable goodness-of-fit of TICS (factor loadings = 0.492–0.814), as well as good internal consistency and invariant structures between Western and Eastern societies, medical and community populations, and across gender and age groups (Duan and Bu, 2017). In the present study, the reliabilities of caring (α = 0.85), inquisitiveness (α = 0.82), and self-control (α = 0.78) were excellent.

#### Strength Use Scale (SUS)

Strength use was assessed by the 14-item SUS (Govindji and Linley, 2007). The scale was preceded by the following prompt: “The following questions ask you about your strengths, that is, the things that you are able to do well or do best.” Responses from the SUS (e.g., “I am able to use my strengths in lots of different situations”) were all made on a seven-point Likert scale (1 = strongly disagree; 7 = strongly agree). High mean scores in each scale indicate high levels of strength use. The goodness-of-fit indices of the revised Chinese version were satisfied among adolescents (Duan et al., 2018b), with the standardized factor loadings ranging from 0.52 to 0.91. In the present study, the SUS Cronbach alpha was 0.96.

#### Educational Outcomes

Academic achievement was assessed through the final exam grades. The official school records provided final exam grades for eight major academic courses, including Chinese, Mathematics, English, History, Geography, Biology, Physics, and Chemistry. The middle schools follow the normal grading systems in China. All students are required to attend the formal final exams. A student who is absent from the exam could make up the test in a later period. We averaged the final exam grades of the participants in the said courses to reflect academic achievements. The level of eudaimonic well-being was assessed by an eight-item flourishing scale (FS), which describes human functioning from a broad perspective (i.e., competence, engagement, meaning and purpose, optimism, self-acceptance, supportive relationships, well-being of others, and being respected) (Diener et al., 2010; Duan and Xie, 2019). A seven-point Likert scale (1 = “strongly disagree”; 7 = “strongly agree”) was adopted to rate items such as “I am engaged and interested in my daily activities.” The mean score of the entire scale indicates the overall eudaimonic well-being. The goodness-of-fit indexes indicated that the single-factor model of the FS adequately fits the total, male, and female samples among adolescents, and the standardized factor loadings are higher than 0.56 (Duan and Xie, 2019). The solid single-factor structure, convergent, and discriminant validity of the FS likewise proved solid among the Chinese community (Tang et al., 2016). In the present study, Cronbach’s alpha was 0.93.

### Analytic Strategy

The means, standard deviation (SD), McDonald’s omega (ω), and Peterson’s coefficient inter-correlations were calculated for all variables using JASP 0.8.1.2. The three character strengths and strength use were positively related to students’ academic achievements and eudaimonic well-being. Subsequently, two regression analyses were performed to examine the relative contributions of the character strengths (caring, inquisitiveness, and self-control) and strength use to educational outcomes (i.e., academic achievement and eudaimonic well-being). In the first regression with academic achievement as the dependent variable, gender and age were set as the control variables in Step 1, followed by the three strengths in Step 2 and strength use in Step 3. Similarly, the second regression replicated the steps, but with the dependent variable as eudaimonic well-being. Finally, on the basis of the regression results, two mediation models were constructed using Mplus 7.0 to examine the direct and indirect effects of strength use between the abovementioned three character strengths and academic achievements/eudaimonic well-being. Maximum likelihood (ML) estimation was adopted. To evaluate the model fit, we used the comparative fit index (CFI > 0.90), Tucker–Lewis index (TLI > 0.90), standardized root mean squared residual (SRMR < 0.80), and root mean square error of approximation (RMSEA < 0.08) to indicate close and reasonable fit (Hu and Bentler, 1999).

## Results

### Descriptive Statistics and Correlations

Table 1 reports the descriptive and correlation statistics. As expected, character strengths and strength use were positively related to students’ academic achievements and eudaimonic well-being (*r* = 0.17–0.79, *p* < 0.001). Moreover, character strengths were positively related to strength use (*r* = 0.32–0.55, *p* < 0.001).

**TABLE 1:** Descriptive and correlations statistics (*n* = 349).

	**1**	**2**	**3**	**4**	**5**	**6**
1 Strengths use	−	0.51^∗∗∗^	0.32^∗∗∗^	0.55^∗∗∗^	0.60^∗∗∗^	0.30^∗∗∗^
2 Self-control		−	0.53^∗∗∗^	0.63^∗∗∗^	0.51^∗∗∗^	0.27^∗∗∗^
3 Caring			−	0.51^∗∗∗^	0.56^∗∗∗^	0.17^∗∗∗^
4 Inquisitiveness				−	0.48^∗∗∗^	0.27^∗∗∗^
5 Well-being					−	0.29^∗∗∗^
6 Academic achievement						−
Mean	4.73	3.42	4.02	3.49	5.30	78.21
SD	1.01	0.64	0.64	0.66	1.06	8.82
Cronbach’s alpha	0.96	0.78	0.85	0.82	0.93	−

*^∗∗∗^*p* < 0.001.*

### Regressions

Table 2 summarizes the regression results. Gender and age could not significantly explain the variance with academic achievements or eudaimonic well-being. Specifically, when predicting academic achievements, only inquisitiveness and self-control were positive predictors, which explained 9.20% of variances. Strength use became the only significant predictor after entering Step 3, which additionally contributed 2.30% variances to academic achievements. When predicting eudaimonic well-being, all three character strengths significantly explained 38.90% of variances beyond demographic variables. After entering strength use in Step 3, caring strength remained a significant predictor of eudaimonic well-being, whereas inquisitiveness and self-control were not. In general, strength use contributed additional explanations to academic achievements (Δ*R*^2^ = 0.023, *p* < 0.001) and eudaimonic well-being (Δ*R*^2^ = 0.133, *p* < 0.001) beyond character strengths.

**TABLE 2:** Regression results on academic achievement and flourishing (*n* = 349).

**Independent variables**	**Dependent variables: academic achievement**		
	**Beta (*t*)**		
	**Step 1**	**Step 2**	**Step 3**
Gender	−0.053 (−0.964)	−0.064 (0.232)	−0.050 (−0.937)
Age	−0.049 (−0.878)	−0.047 (0.372)	−0.045 (−0.862)
Caring		0.004 (0.950)	0.010 (0.165)
Inquisitiveness		0.170^∗^(0.014^)^	0.094 (1.294)
Self-control		00.156^∗^(0.026)	0.105 (1.478)
Strengths use			0.188^∗∗^(2.963)
*R^2^ (F)*	0.004 (0.683)	0.092 (6.922^∗∗∗^)	0.114 (7.362^∗∗∗^)
Δ *R^2^* (Δ *F)*		0.088 (11.041^∗∗∗^)	0.023 (8.778^∗∗^)
**Independent variables**	**Dependent variables: wellbeing**		
Gender	−0.002 (−0.029)	−0.011 (−0.252)	0.023 (0.605)
Age	0.003 (0.045)	0.001 (0.020)	0.006 (0.160)
Caring		0.372^∗∗∗^(7.193)	0.387^∗∗∗^(8.454)
Inquisitiveness		0.154^∗∗^(2.730)	−0.030 (−0.553)
Self-control		0.212^∗∗∗^(3.705)	0.088 (1.695)
Strengths use			0.455^∗∗∗^(9.782)
*R*^2^ (*F*)	<0.001 (0.002)	0.389 (43.754^∗∗∗^)	0.523 (62.476^∗∗∗^)
Δ *R^2^* (Δ*F)*		0.389 (72.921^∗∗∗^)	0.133 (95.692^∗∗∗^)

*^∗^*p* < 0.05, ^∗∗^*p* < 0.01, ^∗∗∗^*p* < 0.001.*

### Structural Equation Models to Test Mediation Effects

The abovementioned results showed that the specific dimensions of character strengths have different effects on specific outcome variables. Hence, two initial structural equation models (SEM) were constructed accordingly. In Model 1, inquisitiveness and self-control were used as correlated predictors, strength use was used as a mediator, and academic achievement was used as an outcome. In Model 2, the three character strengths were used as correlated predictors, strength use was used as a mediator, and eudaimonic well-being was used as an outcome. The fit indices of the initial Model 1 (χ^2^ = 842.026, df = 269, SRMR = 0.042, RMSEA = 0.078, CFI = 0.904, TLI = 0.893) and Model 2 (χ^2^ = 1883.559, df = 619, SRMR = 0.058, RMSEA = 0.077, CFI = 0.868, TLI = 0.858) were acceptable. When the direct paths from dependent variables to academic achievements/eudaimonic well-being were non-significant, such paths were removed from the model, and the parsimonious version was re-run to include only significant paths. The final Model 1 (χ^2^ = 849.433, df = 271, SRMR = 0.044, RMSEA = 0.078, CFI = 0.903, TLI = 0.893) and Model 2 (χ^2^ = 1891.069, df = 622, SRMR = 0.058, RMSEA = 0.076, CFI = 0.868, TLI = 0.858) were constructed with an unchanged goodness-of-fit.

**FIGURE 1:**
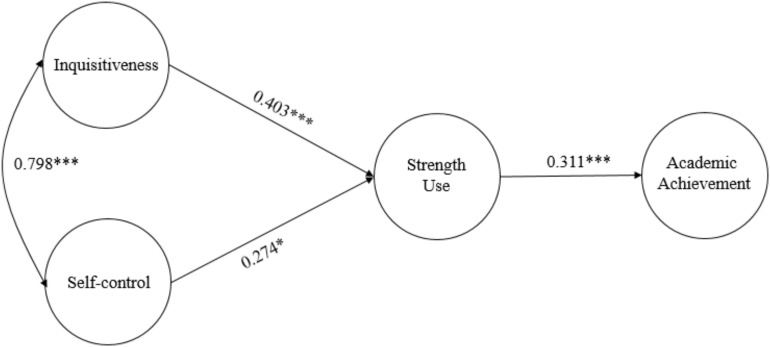
Strengths use mediates the relationship between character strengths and academic achievement (Model 1). ^∗^*p* < 0.05, ^∗∗∗^*p* < 0.001.

Figure 1 shows the parameter estimates of Model 1. The direct effects of character strengths on academic achievements were non-significant (*p* > 0.05), whereas the indirect effects of inquisitiveness (β = 0.125, SE = 0.039, *p* < 0.001) and self-control (β = 0.085, SE = 0.036, *p* < 0.05) via strength use on academic achievements were significant.

**FIGURE 2:**
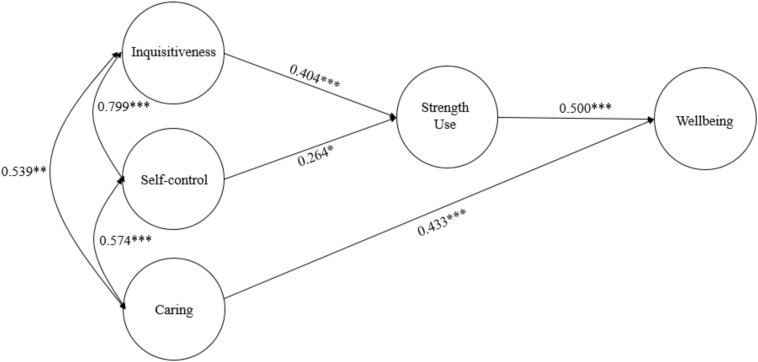
Strengths use mediates the relationship between character strengths and well-being (Model 2). ^∗^*p* < 0.05, ^∗∗^*p* < 0.01, ^∗∗∗^*p* < 0.001.

Figure 2 exhibits the parameter estimates of Model 2. The direct effect of caring on eudaimonic well-being was significant (β = 0.433, SE = 0.040, *p* < 0.001). The indirect effects of inquisitiveness (β = 0.202, SE = 0.056, *p* < 0.001) and self-control (β = 0.132, SE = 0.055, *p* < 0.05) via strength use on eudaimonic well-being were significant.

## Discussion

The present study mainly aimed to systematically explore the profiles of strengths that highly relate to academic achievements and eudaimonic well-being and then test the mediation effect of strength use. As expected, the three character strengths (i.e., caring, self-control, and inquisitiveness) showed significant positive correlations with academic achievements and eudaimonic well-being. Regressions indicated that inquisitiveness and self-control significantly predicted academic achievements and eudaimonic well-being, but caring significantly predicted eudaimonic well-being rather than academic achievements. The SEM results further indicated that strength use mediated the predictive effects of inquisitiveness and self-control on academic achievements/eudaimonic well-being, whereas caring showed a direct effect on eudaimonic well-being. Overall, these results supported our hypotheses and suggested that strength use could be an internal mechanism between the relationship of character strengths and educational outcomes.

The hierarchical regression models revealed interesting findings. Inquisitiveness and self-control predicted academic achievements and eudaimonic well-being, whereas caring was a stronger predictor of eudaimonic well-being than inquisitiveness and self-control. These findings highlight the different functions of character strengths for educational outcomes. According to the strength model of self-control, students with high self-control love learning and are good at effectively managing study time and directing their efforts toward one area, which promote their academic achievements (Tangney et al., 2004; Duckworth and Seligman, 2006). Inquisitiveness, as a form of intrinsic motivation, was associated with curiosity, bravery, zest, and reflected intellectual endeavors (McGrath, 2015; Oudeyer et al., 2016). This trait is regarded as the important pillar of academic achievements (von Stumm et al., 2011). Caring strengths are those that maintain good relations toward others, and studies have shown that these strengths are more strongly connected to eudaimonic well-being than intellectual strengths (similar to inquisitiveness strengths) and strengths of restraints (similar to self-control strengths) (Martinez-Marti and Ruch, 2017).

The SEM results revealed that strength use mediated the relationships between inquisitiveness/self-control and academic achievements/eudaimonic well-being. The self-concordant model (Sheldon and Elliot, 1999) suggested that attaining goals that are self-concordant would obtain more beneficial outcomes (e.g., achievement and eudaimonic well-being) than those that are not. People who use their character strengths would stay true to their interests and values, which would help set self-concordant goals. For example, in a study of university students, using signature strengths offered a reliable pathway of setting and attaining self-concordant goals, which in turn promoted positive affect and eudaimonic well-being (Linley et al., 2010). A new study used the within-person approach by recording fluctuations in the strength use of 87 Norwegian naval cadets over the course of 30 working days. Individuals were found to experience a peak in positive affect and work engagement on days when they used their core strengths (Bakker et al., 2019).

Unexpectedly, in the present study, strength use did not play a role in the relationship between caring and eudaimonic well-being. The conceptual overlaps between strengths and values could provide a tentative explanation. This overlap can lead people to the overestimation of the similarity of others’ actions and thoughts to their own, and the underestimation of their own prowess may lead to blindness regarding their own strengths, such as kindness, curiosity, and bravery, especially for people whose values have evolved (Biswas-Diener et al., 2011). Caring is deeply embedded in Chinese culture. Chinese people could obtain a high level of well-being by adapting to social and ethical norms (Suh et al., 1998), such as making their significant others (e.g., family member) feel happy (i.e., caring) (Ho, 2010). For certain Chinese, caring is viewed as the “right thing” rather than an “extraordinary thing.” As such, they do not realize that caring is a character strength and therefore do not consider actions of caring as strength use. Similarly, considering the role and function of strength knowledge is valuable. What needs acknowledgment is that the overcoming of strength blindness could be beneficial in improving individuals’ self-efficacy and self-confidence, which in turn could affect individual well-being (Waters, 2015). Self-determination theory likewise indicates that the fulfillment of psychological needs for competence enhances personal developments and well-being (Ryan and Deci, 2000, 2001). Strength use could be practiced and developed over time and then deliver sustainable benefits, but strength knowledge could move from “no” (strengths knowledge = 0) to “have” (strengths knowledge = 1) and then deliver an initial benefit (Duan et al., 2018a). Thus, as suggested by Duan et al. (2018a), strength knowledge can be set as a binary variable to investigate its role between character strengths and outcomes in future studies.

This study has several theoretical and practical implications. On the one hand, the study provides evidence on the causal relationship between the three character strengths and academic achievements and eudaimonic well-being, as well as an underlying mechanism of how character strengths contribute to educational outcomes by considering strength use. These results extend the A-E-A model and previous research on the association between character strengths and educational outcomes. Most importantly, this study points out that strength use plays a key role in the A-E-A model, which proves the effectiveness of strength use in strength-based interventions. On the other hand, the findings demonstrated the sights of character strengths and positive education for current and future educational programs and provided practical implications for teachers and practitioners who wish to enhance students’ academic achievements and eudaimonic well-being. Encouraging students to use their strengths is an effective guiding strategy for educators, teachers, and school coaches, and strategies for different goals must be targeted. For instance, if the intervention aims to promote students’ academic achievements, cultivating their use of inquisitiveness and self-control could be a better choice than cultivating their use of caring. Moreover, teachers and practitioners could likewise benefit from a positive education program. Hence, the results of this study likewise provide enlightenment for strength-based interventions conducted for teachers and practitioners.

### Limitations and Future Directions

Despite these interesting and meaningful implications, several limitations of the present study must be highlighted. First, although character strengths are important factors in the positive educational context, their importance must not be over-expanded. Spengler et al. (2016) found that the characteristics, intelligence quotient, and perception of the academic ability of students likewise predicted the stable part of their grades. Second, the present study does not address the mechanisms of how strength use affects educational outcomes and the other potential mechanisms between character strengths and educational outcomes. For instance, Allan and Duffy (2014) found that the presence of calling moderates the relationship between strength use and satisfaction in the life and academic domains. Gillham et al. (2011) argued that other-directed (similar to caring) strengths could predict high well-being via the mediation effect of social support. Hence, more elements could be incorporated into the study of the educational context to reveal other influencing factors in favor of valued educational outcomes in the future. Third, the samples were collected in selected institutions, the inherent nature of which may influence the results. For instance, certain schools would substantially focus on academic achievements, whereas others may remarkably focus on the development of learned quality. In the future, a great diversity of samples and different school levels are necessary to ensure generalizable findings. Fourth, although the present study has a smaller sample size than the ideal, which is a size-to-parameter ratio of 20:1 (Kline, 2010), it met the less ideal criterion (i.e., size-to-parameter ratio between 10:1 and 15:1) recommended by Thompson (2000). Previous studies, such as those conducted by Lowe (2018) and Feldt et al. (2015), have adopted such a sample size for model fitting. Therefore, our current sample size can be justified. However, increasing the sample size in the future is worth taking to facilitate results in further research.

## Conclusion

The character strengths of inquisitiveness and self-control predicted academic achievements and eudaimonic well-being, whereas the strength of caring only predicted eudaimonic well-being. Strengths use played a bridge between inquisitiveness/self-control and academic achievements and eudaimonic well-being, whereas caring had a direct effect on eudaimonic well-being. Positive education programs in campuses could be developed through strength-based approaches with the focus on strength use.

## Data Availability

The raw data supporting the conclusions of this manuscript will be made available by the authors, without undue reservation, to any qualified researcher.

## Ethics Statement

The studies involving human participants were reviewed and approved by the Human Subjects Ethics Sub-Committee of the Department of Sociology, Wuhan University. The patients/participants provided their written informed consent to participate in this study.

## Author Contributions

XT designed the study, analyzed the data, wrote the manuscript, and prepared the submission materials. WD and XC collected the data and comment on the original version. YL and WM helped to revise the manuscript and conducted the additional analyses.

## Conflict of Interest Statement

The authors declare that the research was conducted in the absence of any commercial or financial relationships that could be construed as a potential conflict of interest.
